# A Power Spectrum Maps Estimation Algorithm Based on Generative Adversarial Networks for Underlay Cognitive Radio Networks

**DOI:** 10.3390/s20010311

**Published:** 2020-01-06

**Authors:** Xu Han, Lei Xue, Fucai Shao, Ying Xu

**Affiliations:** 1 Electronic Countermeasure College, National University of Defense Technology, Shushan District, Hefei 230037, China; hanxu17@nudt.edu.cn(X.H.); lei_xue1020@163.com(L.X.); 2 Beijing Military Representative Office, Beijing 100191, China; fucai_shao@126.com

**Keywords:** underlay cognitive radio networks, power spectrum maps estimation, deep learning, generative adversarial networks, image reconstruction

## Abstract

In the underlay cognitive radio networks, the main challenge in detecting the idle radio resources is to estimate the power spectrum maps (PSMs), where the radio propagation characteristics are hard to obtain. For this reason, we propose a novel PSMs estimation algorithm based on the generative adversarial networks (GANs). First, we constructed the PSMs estimation model as a regression model in deep learning. Then, we converted the estimation task into an image reconstruction task by image color mapping. We fulfilled the above task by designing an image generator and an image discriminator in the proposed maps’ estimation GANs (MEGANs). The generator is trained to extract the radio propagation characteristics and generate the PSMs images. However, the discriminator is trained to identify the generated images and help to improve the generator’s performance. With the training process of MEGANs, the abilities of the generator and the discriminator are enhanced continually until reaching a balance, which means a high-accuracy PSMs estimation is achieved. The proposed MEGANs algorithm learns and utilizes accurate radio propagation features from the training process rather than making direct imprecise or biased propagation assumptions as in the traditional methods. Simulation results demonstrate that the MEGANs algorithm provides a more accurate estimation performance than the conventional methods.

## 1. Introduction

As wireless communication technologies continue to grow, radio resources are facing huge demands. Cognitive radio (CR) technology is an important technology in wireless communications. To achieve better exploitation of the radio resources, the CR transceivers intelligently change their transmitting parameters based on detecting and utilizing the radio “white holes” [1,2]. The term “white holes” refers to the unused radio resources in frequency, space, time domains, etc. [3,4].

The cognitive radio networks (CRNs) play a fundamental role in the applications of the CR technology. There are two main components in the cognitive radio networks: the primary users (PUs) and the secondary users (SUs) [5]. The PUs network is a licensed network that initially owns the spectrum resources. However, the SUs network refers to the unlicensed network that aims to access the licensed spectrum dynamically. CRNs can improve radio resources’ utilization by allowing SUs to opportunistically access a licensed band, provided that the PUs is absent; i.e., the utilization of the radio “white holes”.

**Figure 1:**
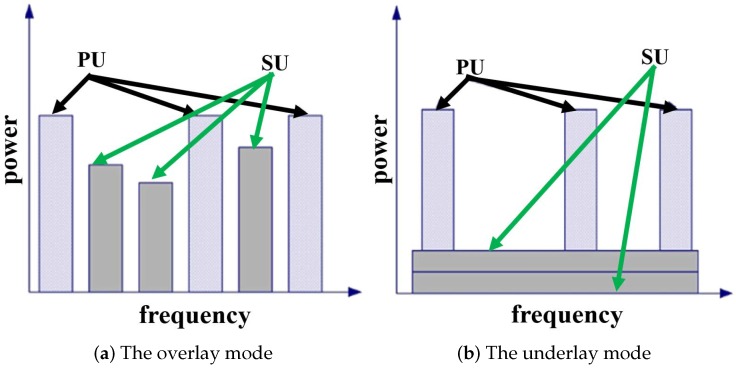
The overlay and underlay modes of cognitive radio networks (CRNs).

CRNs typically work on two main modes: the overlay mode and the underlay mode, as shown in Figure 1. In the overlay mode, the SUs are allowed to access the spectrum holes, which are unoccupied by PUs [6]. They directly target temporal spectrum “white holes” by allowing secondary users to identify and exploit instantaneous spectrum availability in a non-intrusive manner. The “white holes” in the overlay mode are mainly about the unused radio resources in the frequency domain. The common practice is, if several SUs discover that a PU is using a certain frequency, the whole SU network will avoid using that frequency. However, CRNs usually spread over a large region relative to the range of PUs. To further enhance the utilization of radio resources, secondary users can safely use the same frequency outside the range of PUs, which is the core idea of the underlay CR networks.

Regarding the underlay CR networks, which are also known as the spatial reuse CRNs, CR transmissions are permitted on the condition that the interference from SUs is under certain limits and does not degrade the quality of service (QoS) of PUs due to the attenuation in the propagation paths [7]. This approach imposes severe restrictions on the transmission power of SUs. The “white holes” in the underlay mode are mainly about the unused radio resources in the spatial domain. By employing efficient radio management techniques, the underlay CRNs can significantly increase the spatial efficiency of the spectrum.

To achieve the spatial reuse of the radio resources, SUs have to sense the spectrum and exploit the spatial “white holes” in the CRNs’ region. Herein, the power spectrum maps (PSMs) can be used as a powerful tool to determine the PUs’ signals across a finite geographical area. Based on the estimation result of the power spectrum maps, we can obtain the distribution of the signal strength and estimate the spectrum utilization in a particular region. The power spectrum map can be seen as a visible cartography of the power spectrum. In addition, it is a developing technology that visually overlays power spectrum information on a map, enabling rapid frequency deconfliction and maximizing the utilization of the available spectrum. Many schemes are focused on the applications of the power spectrum maps. For example, in [8], the authors exploit the TV white space for device-to-device (D2D) communications with the aid of the existing cellular infrastructure. The power spectrum maps provide a service for the D2D link to determine its maximum permitted emission power in an unlicensed digital TV band, which enables opportunistic transmission across the available channels. To construct and manage a spectrum database to obtain the temporal and spatial spectrum availability information, the authors in [9] propose a joint tensor completion and prediction scheme for multi-dimensional spectrum map construction. Furthermore, aiming to provide real-time awareness of radio spectrum utilization across time, frequency and geography, DARPA has also launched its advanced RF mapping program, known as Radio Map [10].

As for the underlay CRNs, the PSMs portray the primary users’ power distribution in frequency and space [11,12]. Knowing the power spectrum (PS) at any location is particularly useful in wide-area CR networks, where the power transmitted by PUs reaches only a small subset of SUs [13]. Estimating and utilizing the PSMs allow remote SUs to reuse the idle bands dynamically. The power spectrum maps also enable SUs to adapt their transmitting power to minimize the interference to primary users. The PSMs estimation has been identified as a key functionality to ensure that SUs do not interfere with PUs.

**Figure 2:**
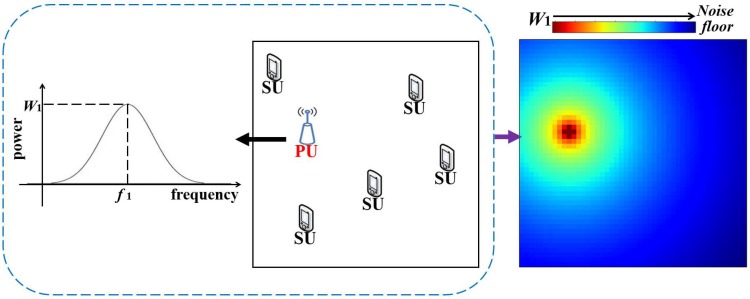
From left to right: The power spectrum of the primary user (PU); the distribution of the transmitting PU and receiving secondary users (SUs); the power spectrum map at frequency $f_{1}$.

As shown in Figure 2, the general setup for estimating the power spectrum maps includes several receiving SUs and transmitting PUs, which are uniformly distributed in the target region. The receiving SUs are willing to cooperate in estimating the power spectrum maps of the target region under an additive white Gaussian noise with a known variance (i.e., the noise floor in Figure 2). Suppose that the number of SUs, their locations and the receiving power spectrum are known, but the number of PUs, their locations or the transmitting power spectrum are unknown. Our task is the following: using the above-known parameters to estimate the power spectrum at any location; i.e., the power spectrum maps of the target region.

The above task is actually undetermined. In fact, there is an infinite number of PSMs functions which can satisfy the known parameters; i.e., there are numerous solutions for the above task. To reduce the solution space of the estimation task, initial efforts have been made for the PSMs estimation by utilizing the prior information or assumptions of the radio environment of the target region. Traditional methods for the estimation task include spatial interpolation algorithms and the basis expansion (BP) algorithm.

The conventional spatial interpolation algorithms include the Kriging interpolation [14,15] and the inverse distance weighted (IDW) interpolation [16,17]. The Kriging interpolation algorithm originates from the geological mineral reserves calculation task. As for the PSMs estimation task, the algorithm assumes that the unknown power spectrum values can be estimated with weighted linear combinations of the available power spectrum values, expressed through the semi-variogram, which quantifies the relationships between the average field value differences of different locations and the distances separating them. We can regard the semi-variogram (e.g., linear variogram, exponential variogram, etc.) as spatial characteristics assumptions of the target region [18].

The IDW interpolation algorithm assumes that the power spectrum only depends on the distance $d_{t}$ between the interpolation node and the receiving SU. The power value $p_{v}$ of the inverse distance (i.e., ${\left(\frac{1}{d_{t}}\right)}^{p_{v}}$) controls the influences of the known nodes on the interpolation node. The IDW interpolation algorithm is not related to any actual physical process. It is difficult to determine whether the specific power value $p_{v}$ is appropriate or not.

Regarding the basis expansion algorithm [19,20], the authors exploit the sparsity in frequency and space to establish the basis expansion model. The radio environment prior information is utilized by directly adopting an approximate radio propagation function (the Okumura–Hata model, the inverse polynomial law model, etc.) of the target region. In addition, the basis expansion algorithm also assumes that the primary users lie in several candidate locations.

All the algorithms mentioned above are effective in a particular environment. However, the actual radio environment is often complex. Inappropriate or biased assumptions may lead to inaccurate PSMs estimation.

Deep learning, as the core technology of the artificial intelligence (AI), has unique advantages in power spectrum map estimation. By learning and adjusting the deep neural network’s parameters, the deep learning methods can achieve infinite approximations of any complicated functions. Through extracting the essential characteristics of the radio environment, we can collect all kinds of states and prior information for CRNs by the deep neural network, which makes the PSMs’ estimation more precise and intelligent. Estimating PSMs from secondary users’ PS measurements is actually a regression task. Generative adversarial networks (GANs) are recently introduced as a powerful framework to handle regression problems in deep learning [21]. There are two main components in the generative adversarial networks: the generator (*G*) and the discriminator (*D*). The strategy of GANs is defining a game between the generator and the discriminator [22]. The generator is trained to generate a high-accuracy estimation of the PSMs and fool the discriminator; the discriminator is trained to decide if the generated PSMs are true or false. Generative adversarial networks are widely used in computer vision and have achieved good performance in object detection, image super-resolution and so on. However, using GANs to estimate power spectrum maps has not been reported until now.

In this paper, we propose a novel GANs-based power spectrum maps estimation algorithm named maps estimation GANs (MEGANs) for underlay CR networks. First, we analyze and construct the PSMs model of the target region as a regression model in deep learning. Once the power spectrum maps matrices have been normalized and transformed by color mapping, the colored PSMs images are constructed. Through the color mapping process, we convert the PSMs estimation task into an image reconstruction task. We fulfill the above task through constructing an image generator and an image discriminator in the proposed MEGANs. We design the generator based on the analogy of auto-encoders, which is used to exploit the training data set and learn abstract radio propagation features of the target region. Regarding the discriminator, we utilize a deep convolutional structure to identify the generated PSMs images and help the generator to improve the PSMs estimation performance. Finally, as verified by simulations, the proposed MEGANs algorithm provides a more accurate PSMs estimation performance than the conventional methods.

The original contributions of our work are as follows:

- Through the process of color mapping, we converted the PSMs estimation task into an image reconstruction task and accomplished it by generative adversarial networks. Utilizing GANs for the PSMs estimation task has not been reported until now.
- With the analogy of auto-encoders and the deep convolutional structure, we designed the generator and the discriminator of the proposed MEGANs to estimate the PSMs and enhance the estimation accuracy.
- We extracted and utilized the radio environment features of the target region from the training process by the proposed MEGANs algorithm rather than the traditional methods, which make direct biased or imprecise assumptions about the spatial propagation characteristics. The MEGANs algorithm provides a more accurate estimation performance than the traditional methods, as verified by simulations.

The rest of the paper is organized as follows. In Section 2, we analyze and construct the power spectrum maps model. In Section 3, we propose a PSMs estimation algorithm for underlay CR networks based on MEGANs. In Section 4, we describe simulation experiments and analyze the results. Finally, we conclude the research findings in Section 5. In addition, the summary of acronyms used in our paper is listed in Table 1.

## 2. Power Spectrum Maps Model

We suppose that there are $N_{R}$ receiving SUs and $N_{T}$ transmitting PUs, which are uniformly distributed in the square target region ***S***. The receiving SUs are willing to cooperate in estimating power spectrum maps of the target region under an additive white Gaussian noise with a known variance. Let ${\left\{{\;\;\;\Phi \;\;\;}_{i}\left(f\right)\right\}}_{i=1}^{N_{R}}$ denote the receiving power spectrum of SUs located at positions ${\left\{(x_{i},y_{i})\right\}}_{i=1}^{N_{R}}$. Let ${\left\{\Psi_{i}\left(f\right)\right\}}_{i=1}^{N_{T}}$ denote the transmitting power spectrum of PUs located at positions ${\left\{(p_{i},q_{i})\right\}}_{i=1}^{N_{T}}$. Suppose that $N_{R}$, ${\left\{(x_{i},y_{i})\right\}}_{i=1}^{N_{R}}$, ${\left\{{\;\;\;\Phi \;\;\;}_{i}\left(f\right)\right\}}_{i=1}^{N_{R}}$ are known but $N_{T}$, ${\left\{(p_{i},q_{i})\right\}}_{i=1}^{N_{T}}$, ${\left\{\Psi_{i}\left(f\right)\right\}}_{i=1}^{N_{T}}$ are unknown. Let $g_{\left(p,q)\to (x,y\right)}$ be the unknown radio propagation function from the primary user’s location (p,q) to the secondary user’s location (x,y). The known power spectrum relations are shown in Equation (1):

(1) $$\left\{\begin{array}{c} \Phi_{1}\left(f\right)=\sum_{i=1}^{N_{T}}g_{\left(p_{i},q_{i}\right)\to \left(x_{1},y_{1}\right)}\Psi_{i}\left(f\right)+\sigma^{2}\\ \Phi_{2}\left(f\right)=\sum_{i=1}^{N_{T}}g_{\left(p_{i},q_{i}\right)\to \left(x_{2},y_{2}\right)}\Psi_{i}\left(f\right)+\sigma^{2}\\ \;\;\;\vdots \\ \Phi_{N_{R}}\left(f\right)=\sum_{i=1}^{N_{T}}g_{\left(p_{i},q_{i}\right)\to \left(x_{N_{R}},y_{N_{R}}\right)}\Psi_{i}\left(f\right)+\sigma^{2}, \end{array}\right.$$

where $\sigma^{2}$ denotes the variance of the additive white Gaussian noise of the target region.

Let $F_{PS}\left(f;x,y\right)$ denote the power spectrum at location (x,y). It represents the aggregate distribution of the powers across space corresponding to the frequency. The power spectrum maps model is as follows:

(2) $$F_{PS}\left(f;x,y\right)=\sum_{i=1}^{N_{T}}g_{\left(p_{i},q_{i}\right)\to \left(x,y\right)}\Psi_{i}\left(f\right)+\sigma^{2}\;,\forall \left(x,y\right)\in S.$$

As shown in Equation (3), our task is the following: using the above-known power spectrum relations (Equation (1)) and parameters to estimate the power spectrum maps model (Equation (2)); i.e., the power spectrum maps of region ***S***.

(3) $$\begin{array}{c} \begin{array}{cc} \; & F_{PS}\left(f;x,y\right)=\sum_{i=1}^{N_{T}}g_{\left(p_{i},q_{i}\right)\to \left(x,y\right)}\Psi_{i}\left(f\right)+\sigma^{2}\;,\forall \left(x,y\right)\in S\\ s.t.\; & \Phi_{j}\left(f\right)=\sum_{i=1}^{N_{T}}g_{\left(p_{i},q_{i}\right)\to \left(x_{j},y_{j}\right)}\Psi_{i}\left(f\right)+\sigma^{2}\;,j=1,2,...,N_{R}. \end{array} \end{array}$$

The above task is actually undetermined. In fact, there is an infinite number of PSMs functions which can satisfy Equation (1). To reduce the solution space of the estimation task (i.e., Equation (3)), we need to utilize the prior information of the radio environment in the target region. Estimating PSMs from secondary users’ PS measurements is actually a regression task. Generative adversarial networks are powerful frameworks to handle regression problems in deep learning. In this paper, we extract the essential characteristics of the radio environment and utilize them as the prior information to estimate the PSMs based on the proposed MEGANs algorithm.

## 3. MEGANs-Based Power Spectrum Maps Estimation Algorithm

### 3.1. Color Mapping

**Figure 3:**
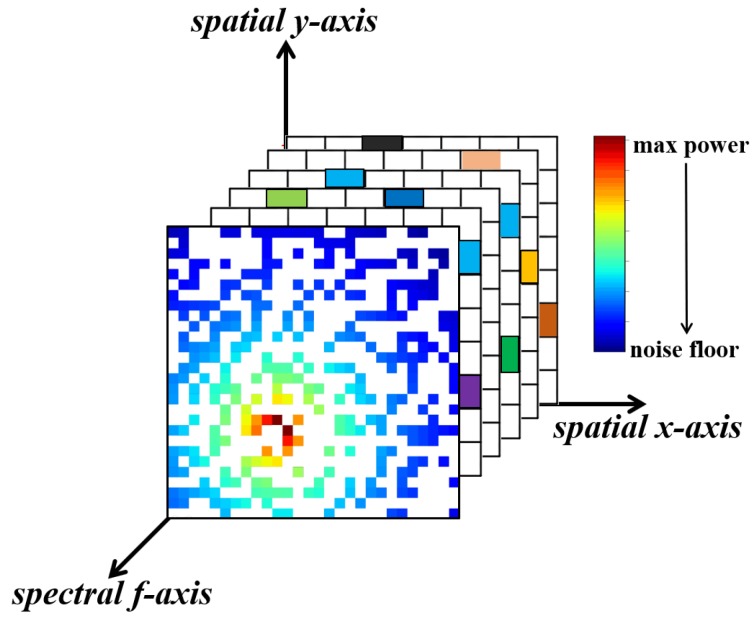
The colored, incomplete power spectrum maps.

Divide the target region ***S*** into N×N grids and suppose each grid has at most one user (a primary user or a secondary user). To facilitate the presentation and the analysis of the MEGANs model, we suppose N=48 and divide region ***S*** into 48×48 grids. We normalize the receiving power spectrum of SUs and color the grids of region ***S*** according to the power values at different frequencies; i.e., map the power components of different frequencies to different colors uniformly, as shown in Figure 3.

The colors of the grids indicate the power value of each location in power spectrum maps. The brighter the color, the bigger the power value is. White squares represent the grids where there are no receiving SUs. The power components in these white squares are exactly our targets that need to be estimated.

Through the process of color mapping, we convert the power spectrum maps estimation task into an image reconstruction task in deep learning. Thus, we can use the powerful regression framework—generative adversarial networks, to handle the PSMs estimation task; i.e., we train the GANs to regress for the missing pixels of the incomplete power spectrum maps images.

### 3.2. Maps Estimation GANs Model

**Figure 4:**
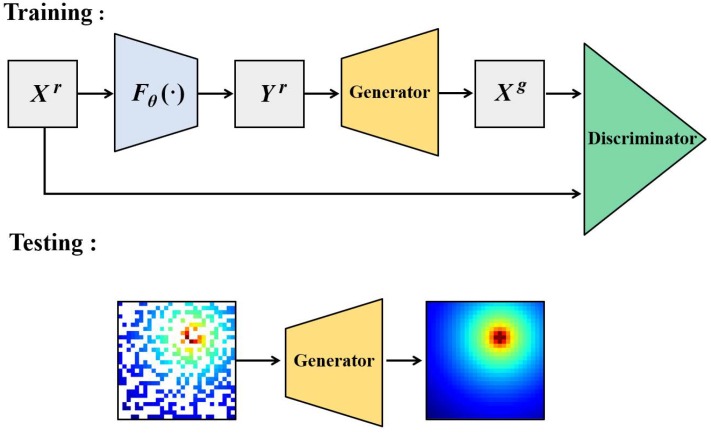
Maps estimation GANs model.

To circumvent the estimation errors from the inaccurate direct assumptions about the prior radio environment information in the conventional methods (Kriging spatial interpolation algorithm, IDW algorithm, etc.), we propose a novel GANs algorithm named maps estimation GANs to estimate the power spectrum maps without any direct spatial characteristic assumptions, as shown in Figure 4.

Throughout, we use superscript “*r*” to denote the real or true power spectrum distribution of the target region, superscript “*g*” for the generated or reconstructed power spectrum distribution from the generator, “*X*” for complete power spectrum maps images and “*Y*” for incomplete power spectrum maps images. Thus, we define the real complete PSMs images as $X^{r}$ and reconstructed complete PSMs images as $X^{g}$, as shown in Figure 4. Let $p_{x}^{r}$ be the underlying distribution of the real complete PSMs images; i.e., $X^{r}∼p_{x}^{r}$. Let $p_{x}^{g}$ be the underlying distribution of the generated complete PSMs images from the generator; i.e., $X^{g}∼p_{x}^{g}$.

We use $F_{\theta }\left(\cdot \right)$ to denote the measurement function that samples lossy measurements from $p_{x}^{r}$. Let $p_{y}^{r}$ be the underlying distribution of the real incomplete PSMs measurements performed on samples from $p_{x}^{r}$; i.e., $F_{\theta }\left(X^{r}\right)=Y^{r}∼p_{y}^{r}$. There are many types of measurement functions to sample incomplete images from $p_{x}^{r}$. We list some of them as follows.

- Random-block-patches (RBPa): We set pixels inside of θ randomly chosen patches to zero. Each patch is with the size of n×n. We can also set the size of each patch according to the target region.
- Random-block-pixels (RBPx): We independently set each pixel of the input PSMs image to zero with probability θ. We assume that θ is uniformly distributed; i.e., $p_{\theta }∼U\left(\alpha ,1\right)$. α should be equal to or less than the proportion of white squares in each incomplete power spectrum map in the testing data set.
- Random-block-patches-pixels (RBPax): This measurement function is the superposition of the RBPa and the RBPx.

The principle that we use in the selection of the measurement functions $F_{\theta }\left(\cdot \right)$ depends on the shape of the white grids in the colored incomplete PSMs images; i.e., the distribution of receiving SUs in the target region. For example, we choose the RBPx measurement function if the receiving SUs are uniformly distributed in the target region ***S***; we choose the RBPax measurement function if there are buildings and streets in the region ***S***. The receiving SUs are uniformly distributed in the streets. The color pixels stand for receiving SUs and the patches stand for buildings where there are no receiving SUs. In fact, we can change and design the measurement functions according to the actual environment and the distribution of the receiving SUs in the target region.

Let *G* denote the generator. We suppose $G\left(Y^{r}\right)=X^{g}$ and $p_{x}^{g}$ be the distribution of $X^{g}$. Our goal is to learn a generator that $p_{x}^{g}$ is equal to or extremely close to $p_{x}^{r}$. The strategy of MEGANs is defining a game between the generator and the discriminator. The generator is trained to generate a high-accuracy estimation of the PSMs and fool the discriminator; the discriminator is trained to decide if the PSMs generated are true or false. Let *D* denote the discriminator. It measures the gap between the real complete PSMs and the reconstructed PSMs from the generator. With the training process of the MEGANs model, the abilities of the generator and the discriminator are enhanced continually until achieving a balance, which means the discriminator cannot tell the reconstructed PSMs images from the real PSMs images; i.e., $p_{x}^{g}$ is an extremely close match to $p_{x}^{r}$.

### 3.3. The Structure of MEGANs

The generative adversarial networks are powerful frameworks to handle the regression problems in deep learning. The two main components of the proposed GANs are the generator and the discriminator.

**Figure 5:**
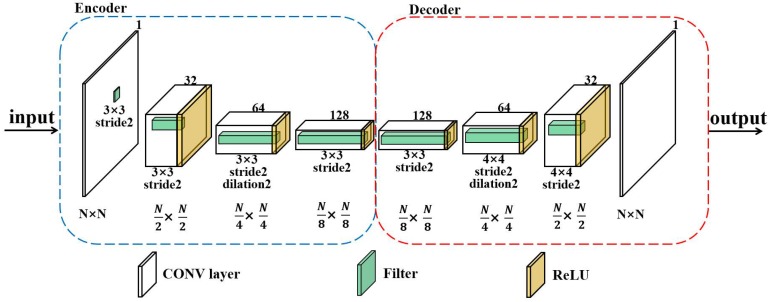
The structure of the generator.

As for the generator, we designed its structure on the analogy of auto-encoders [23] (Figure 5), which was used to exploit the training data set and learn more abstract radio environment features of the target region. We trained the generator to regress for the missing pixels of the incomplete power spectrum maps images. The generator of the MEGANs is closely related to the auto-encoders, as the generator’s structure shares a similar encoder-decoder architecture with them. The proposed generator consists of an encoder to capture the characteristics of an image into latent feature representations and a decoder, which utilizes the representations to produce the missing PSMs’ contents. The generator takes the incomplete PSMs images and tries to reconstruct them by passing through the low-dimensional layer or “bottleneck” layer, with the aim of obtaining feature representations of the input images. The proposed generator not only compresses the image contents but also learns the semantically meaningful representations. We call our model maps estimation GANs, as it can estimate the power spectrum maps from the incomplete PSMs images.

**Figure 6:**
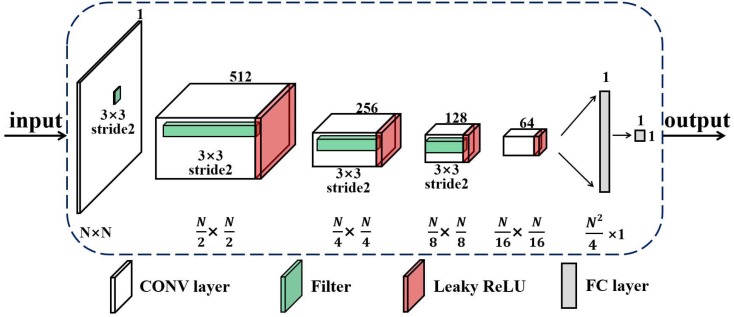
The structure of the discriminator.

Regarding the discriminator, we utilize a deep convolutional neural network (CNN) to identify the PSMs images generated and help the generator to improve the PSMs estimation performance, as shown in Figure 6. The convolutional structure is an important structure in deep learning and has unique advantages in image feature learning [24]. By learning and adjusting the network’s structure, CNN can achieve infinite approximations of any complicated functions. The proposed discriminator directly uses the image as the input of the model without any other image processing, such as the wavelet transformation, high order statistics and so on. The discriminator avoids the complex process of feature extraction and image reconstruction, which overcomes the shortcomings of traditional image processing algorithms. Furthermore, the procedure of weights sharing in CNN significantly reduces the number of training parameters and also decreases the computational complexity [25]. At the same time, the convolution process of the discriminator helps to extract the essential characteristics of images from the training data set and enhances the identification ability towards the reconstructed PSMs images.

The MEGANs model is an incomplete connected neural network, which consists of the input layer, convolutional layer, activation function, fully connected layer and the output layer. The explanations of the above modules are as follows.

- Input layer: As for the generator, we set the incomplete power spectrum maps images $Y^{r}$ as the input layer; i.e., the input of G(·). Regarding the discriminator, we set the complete PSMs images ($X^{r}$ and $X^{g}$) as the input layer; i.e., the input of D(·).
- Convolutional layer (CONV layer): The CONV layer is composed of multiple filters, which are used to implement convolution operations. Every convolution kernel can be regarded as a feature recognizer. The feature map contains the “features” extracted by each filter from the original images. We set several convolutional layers to extract more abstract and deeper features progressively. For example, front convolutional layers can extract low-level features; middle convolutional layers can extract middle-level features; rear convolutional layers can extract high-level features. We extract and compress the features continuously to obtain higher-level features. In short, the original features are condensed step by step and the final features are more reliable. After processing by the activation function $f_{act}\left(\cdot \right)$, the convolutional layers result in the new feature map, as shown in Equation (4).
  (4) $$X^{l}=f_{act}\left(W*X^{l-1}+b\right),$$
  where *X* represents the feature matrix and *l* represents the index of the convolutional layer. We use *W* to denote the weights of the convolution kernel and *b* to denote the convolution offset.
- Activation function: The main effect of the activation function is to provide the nonlinear ability of the network. The neural network can only express linear relationships without the activation function, which makes the whole deep neural network equivalent to the single-layer neural network. Therefore, the deep neural network gets the nonlinear mapping ability only when we use the activation function.
- Fully connected layer (FC layer): The FC layer plays the role of classification and feature combination in the discriminator network. Unlike the CONV layer, which maps the original image data to the local hidden feature space, the FC layer can classify and combine the local features to the globe feature space. After processed by activation functions, the FC layers result in the new feature map, as shown in Equation (5).
  (5) $$x^{l}=f_{act}\left(W^{T}x^{l-1}+b\right),$$
  where *x* represents the feature vector and *T* denotes the matrix transposition.
- Output layer: We set the reconstructed complete power spectrum maps images $X^{g}$ as the output layer of the generator; i.e., the results of $G(Y^{r})$. The discriminator’s outputs are the identification scores of the complete PSMs images ($X^{r}$ and $X^{g}$). The closer the latent distribution $p_{x}^{g}$ of $X^{g}$ is to the real PSMs’ latent distribution $p_{x}^{r}$, the higher identification score the discriminator outputs.

### 3.4. Training Process of MEGANs Algorithm

The MEGANs algorithm includes two processes: the training process and the testing process.

In the training process, the real, complete power spectrum maps images $X^{r}$ in the training data set are sampled by the measurement function $F_{\theta }\left(\cdot \right)$, and then we get the real, incomplete PSMs images $Y^{r}$. We put $Y^{r}$ into the generator to complete the lossy measurements and get the reconstructed complete PSMs images $X^{g}$. Finally, we input $X^{r}$ and $X^{g}$ to the discriminator and get their corresponding identification scores.

During the training process, the generator is trained to generate a high-accuracy estimation of the PSMs and fool the discriminator; i.e., the generator tries to increase the identification scores of $X^{g}$. However, the discriminator is trained to decide if the input images are real or generated; i.e., the discriminator tries to decrease the identification scores of $X^{g}$ and increase the identification scores of $X^{r}$. With the training process of the MEGANs model, the abilities of the generator and the discriminator are enhanced constantly until achieving a balance, which means the discriminator cannot tell the reconstructed PSMs images from the real PSMs images; i.e., $p_{x}^{g}$ is an extremely close match to $p_{x}^{r}$.

The most commonly used training method of the deep neural network is the back-propagation algorithm [26]. We can divide the process of the back-propagation algorithm into two steps: forward data propagation and backward error propagation. In the forward data propagation process, the input layer is used as the initial value, and then the data are pushed forward from the first layer to the last layer. In the backward error propagation process, we correct the weights and offsets of the network according to the errors from the objective function through the supervised learning method.

As mentioned above, the objective function is the key function for the back-propagation algorithm in the training process. To speed up the MEGANs’ training process and enhance the PSMs estimation performance, we consider the white squares in each incomplete PSM’s image as the image noise or redundant information. We use the image denoising regularization (i.e., total variation (TV) norm [27]) as a penalty term to modify the objective function of Wasserstein GAN with gradient penalty (WGAN-GP) [28] as follows:

(6) $$\begin{array}{cc} \min_{G}\max_{D}\;\; & \underset{X^{r}∼p_{x}^{r}}{E}\left[D(X^{r})\right]\\ - & \underset{X^{r}∼p_{x}^{r}}{E}\left[D(G\left(F_{\theta }\left(X^{r}\right)\right))\right]\\ - & \beta \cdot \underset{X^{i}∼p_{x}^{i}}{E}\left[{\left({∥\nabla_{X^{i}}D\left(X^{i}\right)∥}_{2}-1\right)}^{2}\right]\\ + & \lambda \cdot \underset{X^{r}∼p_{x}^{r}}{E}\left[{∥G(F_{\theta }\left(X^{r}\right))∥}_{TV}\right]. \end{array}$$

The third term in Equation (6) is the gradient penalty term in WGAN-GP, which enhances the stability in the training process [28]. β is the coefficient of the gradient penalty term. $X^{i}$ denotes the random linear interpolation of generated samples $X^{g}$ and real samples $X^{r}$.

The fourth term in Equation (6) is the total variation penalty term. ${∥\cdot ∥}_{TV}$ and λ are the total variation norm and its coefficient. The discrete version of the total variation norm for image *M* is defined as follows.

(7) $$\begin{array}{c} {∥M∥}_{TV}=\sum_{i,j}\left({\left(m_{i,j+1}-m_{i,j}\right)}^{2}+{\left(m_{i+1,j}-m_{i,j}\right)}^{2}\right), \end{array}$$

where *M* and $m_{i,j}$ are the input image and the pixel in the *i*th row and *j*th column of the image.

The TV regularization, also known as the total variation denoising, is a process mostly used in digital image processing. It is based on the fact that images with excessive and possibly false details have a high TV norm. Based on the above principle, reducing the TV norm of the images removes unwanted details and preserves important details, such as edges. Therefore, we add the TV regularization to the loss function for searching for a close match to the original PSM images.

In the supervised deep learning method, we use the known paired data in the training set (e.g., the images and their corresponding labels) to train the deep neural network for the classification or the regression task. The proposed MEGANs algorithm is a supervised deep learning method. Estimating PSMs from secondary users’ PS measurements is actually a regression task. The incomplete PSMs’ images and their corresponding, complete PSM images are the known paired data in the training set. In our MEGANs training process, we regard the complete PSM images as the labels in the supervised deep learning. As for the PSM images in the training set, they should be independent and identically distributed as the images in the testing set. Therefore, we should collect the training complete PSMs’ images from the same or similar radio propagation environment as the PSM images in the testing region ***S*** in advance. For example, as discussed in Section 3.1, we can divide a region, which shares the same or similar underlying radio environment characteristics as the target region, into N×N grids and collect the power spectrum measurements in each grid to construct our training set. Furthermore, we can also set the spectrum sensing equipment on the vehicles for collecting the region’s power spectrum measurements to build our training set.

In fact, collecting the training PSMs images or collecting enough PSMs images from the same or similar radio environment is not always an easy task. We should acknowledge that the goal of estimating the high-accuracy power spectrum maps is admittedly very ambitious. However, the PSMs estimation results do not need to be super accurate, but precise enough to identify the spatial “white holes” and the unused bands. This relaxed objective motivated us to choose one or more suitable radio propagation models (the Okumura–Hata model, the inverse polynomial law model, etc.) to generate the training data according to the radio environment in the testing region ***S***. For example, if the testing region ***S*** is the urban area with the quasi smooth terrain, we can use the Okumura–Hata model and/or other similar radio propagation models to generate the training data. In order to help the MEGANs to learn the real underlying radio propagation characteristics and improve the generalization performance, we should use multiple sets of different parameters in the radio propagation models to generate the training data. Furthermore, if we have already collected a small number of real power spectrum maps, adding the generated data into the collected data set to extend our training set is also a good choice.

### 3.5. Testing Process of MEGANs Algorithm

To examine the effectiveness of the MEGANs-based algorithm, it is necessary to test the performance of the proposed method through some testing indicators. We chose three indicators to test the proposed algorithm:

1. The direct visual observation of the PSM images’ reconstruction performance.
2. The power spectrum estimation performance for PUs.
3. The average image reconstruction errors (AIREs) over different numbers of receiving SUs.

The testing process for the direct visual observation of the reconstruction performance was relatively easy. We input the testing incomplete PSM images into the trained generator and then observed the reconstruction performance. It was an intuitive and qualitative testing method.

As for the power spectrum estimation performance, we compared the MEGANs-based estimated power spectrum with the real power spectrum of PUs. The testing result demonstrated the estimation performance for the unused bands based on the proposed algorithm.

Regarding the average image reconstruction errors over different numbers of receiving SUs, we randomly chose *q* frequency points within the transmitting bands of PUs and defined the average image reconstruction errors as shown in Equation (8).

(8) $$\begin{array}{c} A_{err}=\frac{1}{q}\sum_{i=1}^{q}{∥M_{real}^{i}-M_{gen}^{i}∥}_{2} \end{array}$$

where $M_{real}^{i}$ and $M_{gen}^{i}$ are the real complete power spectrum map and the reconstructed complete power spectrum map at the *i* th frequency point. The PSMs are estimated based on the power spectrum measurements from SUs. The more measurements from SUs, the better estimation results from the proposed algorithms. Therefore, the average image reconstruction errors are related to the numbers of receiving SUs. We need to evaluate the $A_{err}$ over different numbers of receiving SUs based on the MEGANs algorithm.

## 4. Simulations

In this section, we describe the test of the estimation performance of the proposed algorithm on the above three indicators in Section 3.5. The simulations were run under the Windows 10 operating system based on Visual Studio Code software. The training and testing of samples was done using a basis of the Pytorch framework. We used the Intel Core i7-8750H processor, and the corresponding graphics card was RTX 2080.

### 4.1. Simulation Settings

In the practical radio environment, the signal is attenuated in a random fashion. The attenuation is mainly caused by the radio propagation loss, the shadow fading, the multi-path effects, etc. We usually construct the radio propagation loss as the deterministic model, e.g., the Okumura-Hata model, the inverse polynomial law model, etc. In addition, the shadow fading and the multi-path effects are estimated by random models, i.e., the log-normal distribution for the shadow fading and the Rayleigh distribution for the multi-path effects, etc. In our simulations, we use the same deterministic radio propagation model as used in [19] rather than random models. The reasons are as follows.

The core purpose of the simulations was to test whether the proposed MEGANs algorithm could learn the accurate radio propagation features from the training process rather than making direct imprecise or biased propagation assumptions, as in the traditional methods. In order to exclude the influence of stochastic factors and intuitively show the propagation features learned from MEGANs, we used the deterministic radio propagation model for the propagation features, which can be easily expressed by a formula. Furthermore, the deterministic propagation model was employed for ease of the comparison experiments with the traditional methods. As for the random attenuation models, the MEGANs can also achieve good estimation performance if the training set and the testing set share the same random distribution of the radio propagation features.

Divide region ***S*** into 48×48 grids and choose the inverse polynomial law model $\gamma_{tr}=\min\left\{1,{\left(d/d_{0}\right)}^{-\alpha }\right\}$ as the radio propagation model for region ***S*** in the simulations [19]. $\gamma_{tr}$ denotes the propagation loss from the transmitter to the receiver. *d* denotes the distance between the transmitter and the receiver. α and $d_{0}$ are the preselected constants depending on the radio propagation environment.

**Figure 7:**
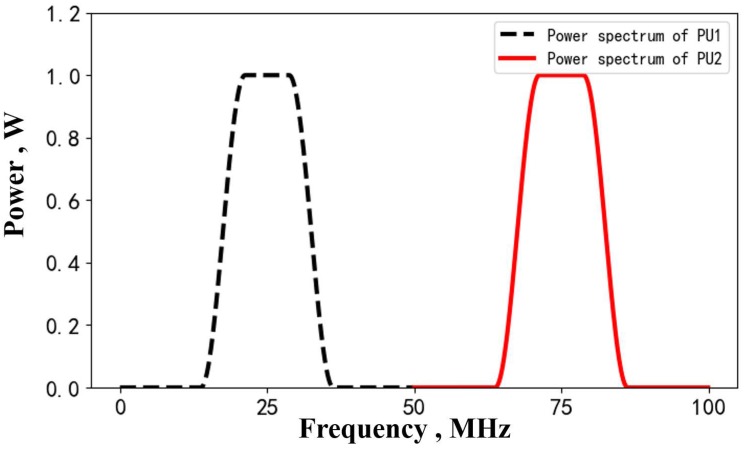
The power spectrum of PU1 and PU2 in the testing data set.

As for the testing set, we supposed α=2 and $d_{0}\;=\;2$ for region ***S***. Suppose there are two active transmitting PUs located at grids (20,18) and (40,35) in the presence of an additive white Gaussian noise with known variance ${\sigma_{1}}^{2}$. The receiving SUs uniformly distribute in region ***S***, accounting for about 15 percent of all grids. Suppose the primary users transmit random signals. We can estimate their corresponding power spectra based on the periodogram method after sampling the PUs’ signals. In our simulation, we directly set the power spectra of PU1 and PU2, as shown in Figure 7, which are centered at 25 and 75 MHz.

As for the training set, we emploedy the same radio propagation model but different preselected constants from the testing set. We generated 30,000 training samples from three sets of environmental propagation parameters: α=3 with $d_{0}\;=\;1$, α=1 with $d_{0}\;=\;3$, and α=1 with $d_{0}\;=\;2$. Each type generated 10,000 PSMs images in the presence of an additive white Gaussian noise with known variance ${\sigma_{2}}^{2}$. The number of PUs transmitters in each map was independently selected from one to five randomly, and the transmitting power was normalized to 1 W. Suppose the receiving SUs uniformly distribute in region ***S*** and use the random-block-pixels measurement model with α=0.15. It should be noted that the training data set does not contain the images in the testing data set.

For the training process, we summarize the learning parameters as follows: the adaptive moment estimation (Adam) algorithm was used for the MEGANs training; the learning rates of the generator and the discriminator were 0.0001; the total variation coefficient was 0.005; the gradient penalty coefficient was 10 [28]; the batch size was 32.

**Figure 8:**
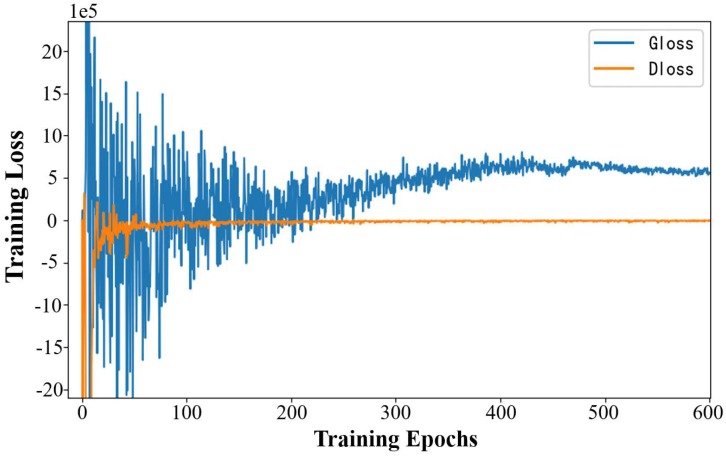
The convergence curves of the generator and the discriminator in the training process. The curve of the generator’s training loss is depicted by the blue solid line. The curve of the discriminator’s training loss is depicted by the orange solid line.

The maximum training epoch in our simulations was set to 600, and it produced sufficient convergence on the training data set. The convergence curves of the generator and the discriminator in the training process are shown in Figure 8.

**Figure 9:**
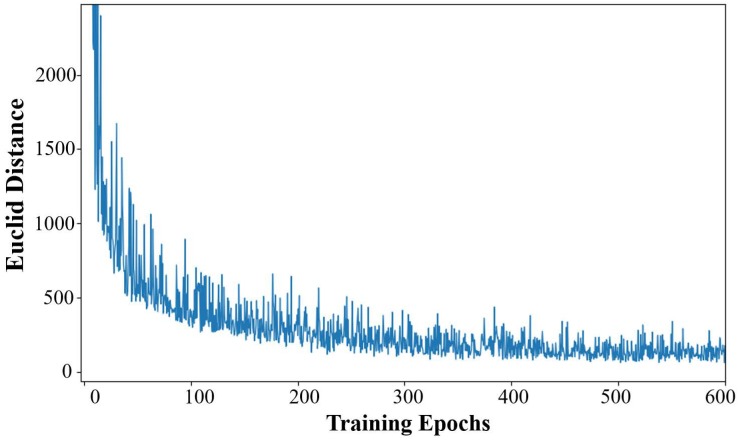
The convergence curve of the Euclid distance $d_{Euc}$ between the generated, complete power spectrum maps (PSMs) and the real, complete PSMs in the training process.

We define the Euclid distance $d_{Euc}$ (i.e., the $l_{2}$ norm) between the generated complete PSMs and the real complete PSMs of the training data set in Equation (9).

(9) $$\begin{array}{c} d_{Euc}=\frac{1}{b}\sum_{j=1}^{b}{∥M_{r}^{j}-M_{g}^{j}∥}_{2} \end{array}$$

where $M_{r}^{j}$ and $M_{g}^{j}$ are the *j*th real complete PSM and the *j*th generated complete PSM in every training batch. *b* stands for the batch size. The convergence curve of $d_{Euc}$ in the training process is shown in Figure 9.

As shown in Figure 8, the training loss of the discriminator converges at around 100 epochs, which means that the discriminator learns the latent distribution of the real PSMs images and gets a strong ability to differentiate between the artificially-generated PSMs images and the real PSMs images. The image reconstruction ability of the generator is weak at this training phase. However, with the training process of MEGANs, the training loss of the generator converges at around 500 epochs, which means that the reconstruction ability of the generator is enhanced constantly until achieving a balance with the discriminator. At this training phase, the discriminator cannot tell the reconstructed PSMs images from the real PSMs images; i.e., $p_{x}^{g}$ is an extremely close match to $p_{x}^{r}$.

The convergence curve of the Euclid distance also proves the above convergence analysis about the training process of MEGANs. As shown in Figure 9, the reconstruction ability of the generator is enhanced constantly and the gaps between the real and generated PSMs images are getting smaller and smaller along with the MEGANs training.

To verify the estimation performance of the proposed algorithm, we compared the MEGANs algorithm with the conventional Kriging interpolation algorithm and the IDW interpolation algorithm on the testing set. As for the simulation of the Kriging algorithm, we denote the Kriging with the linear variogram as L-Krig and the Kriging with the exponential variogram as E-Krig. Additionally, we set the power value $p_{v}=2$ of the inverse distance in IDW interpolation. We did not test the estimation performance of the basis expansion algorithm because the assumptions in BP algorithm are beyond practice and hard to achieve in the actual complex radio environment; e.g., the assumptions of the known radio propagation model, the primary users’ candidate locations and the sparsity in frequency and space [19].

### 4.2. Simulation Results

We tested the estimation performance of the proposed MEGANs algorithm in three aspects: (1) the direct visual observation of the reconstruction performance; (2) the power spectrum estimation performance for PUs; (3) the average image reconstruction errors over different numbers of receiving SUs.

(1) The direct visual observation of the PSMs images reconstruction performance.

**Figure 10:**
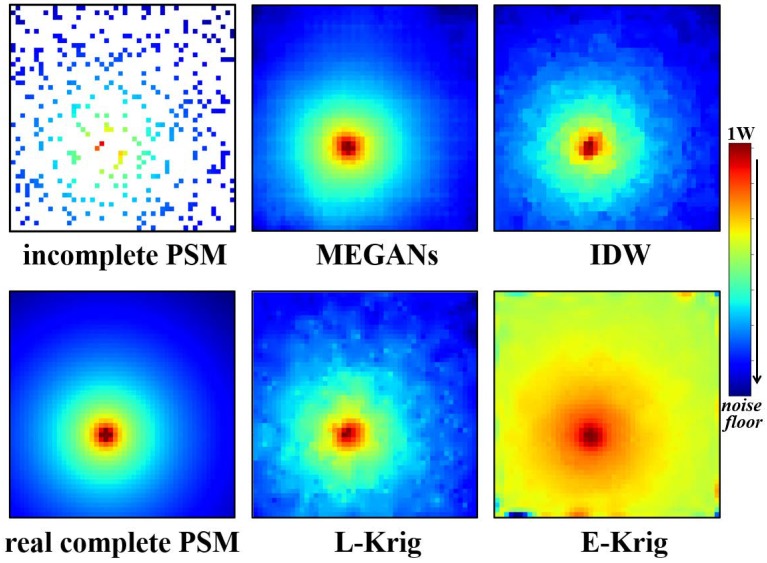
The incomplete PSMs images, MEGANs, IDW interpolation, linear Kriging, exponential Kriging PSMs estimations and the real, complete PSMs images for PU1 at 25 MHz.

**Figure 11:**
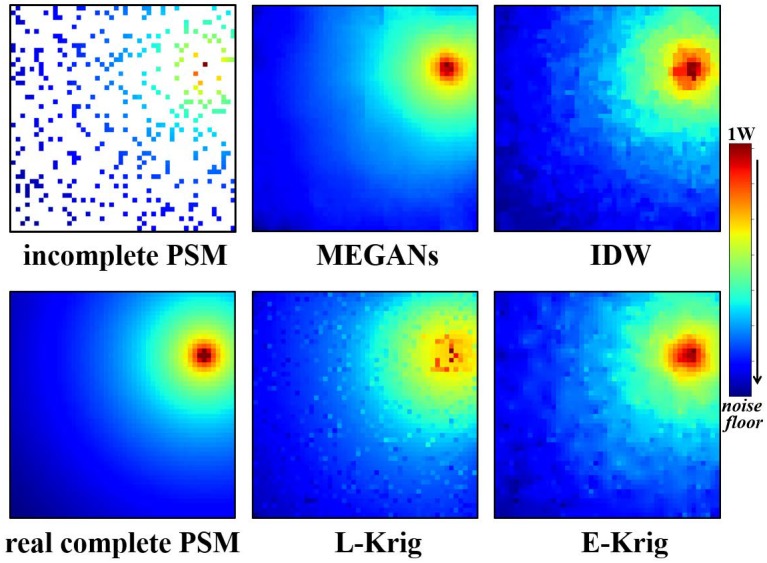
The incomplete PSMs images, MEGANs, IDW interpolation, linear Kriging, exponential Kriging PSMs estimations and the real, complete PSMs images for PU2 at 75 MHz.

After 600 training epochs, we input incomplete PSMs images of the testing data set into the trained generator. The PSMs estimation results for PU1 at 25 MHz and PU2 at 75 MHz are shown in Figure 10 and Figure 11.

Compared with the real, complete PSMs images, the MEGANs algorithm outperforms the Kriging algorithm and the IDW algorithm from the direct visual observation in the estimation results of PU1 and PU2, especially in the area near the PUs. Furthermore, the exponential Kriging algorithm performs poorly for PU1 at 25 MHz because the variogram assumption is of significant deviation from the real data distribution. Furthermore, the reconstructed PSMs images from the proposed MEGANs algorithm are smoother because of the total variation penalty term that we added into the training objective function in Equation (6).

(2) The power spectrum estimation performance for PUs.

**Figure 12:**
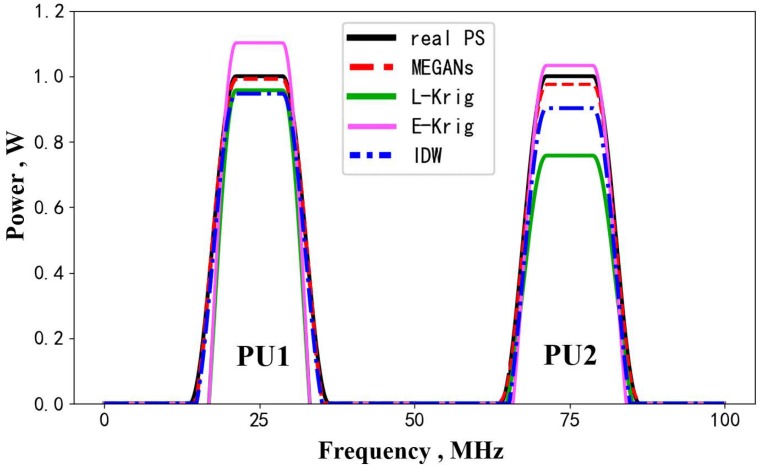
The power spectrum estimation results for PU1 and PU2.

The power spectra transmitted by PU1 and PU2 are centered at 25 and 75 MHz. They are depicted by the black solid line in Figure 12. Compared with the real power spectrum 1 and power spectrum 2, Figure 12 shows that the MEGANs algorithm has a better estimation performance than IDW interpolation algorithm, linear Kriging and exponential Kriging.

The IDW interpolation algorithm assumes that the power spectrum only depends on the distance $d_{t}$ between the interpolation node and the receiving SU. The power value $p_{v}$ of the inverse distance (i.e., ${\left(\frac{1}{d_{t}}\right)}^{p_{v}}$) controls the influences of the known nodes on the interpolation node. Inaccurate settings of the power value $p_{v}$ lead to imprecise PSMs estimation results, as shown in Figure 12. In fact, it is quite difficult to determine whether the specific power value $p_{v}$ is appropriate or not.

As for the above two Kriging algorithms in Figure 12, the gap between the real power spectrum and the estimation results from Kriging algorithm can be explained by the fact that there will always be inaccurate or biased spatial characteristics assumptions (i.e., the variogram assumptions of Kriging) for the complex radio environment of the testing region. However, the spatial characteristics of the radio propagation environments are the key factor for the PSMs estimation.

The proposed MEGANs algorithm has a better estimation performance because we do not directly assume the prior information of the radio environment but train a generator network to learn the power distribution characteristics in region ***S*** from the training data set. It should be noted that the training set does not contain the testing set images, as mentioned in Section 4.1. Thus, the trained MEGANs has a good generalization performance for the testing data set, as verified in our simulations.

(3) The average image reconstruction errors (AIREs) over different numbers of receiving SUs.

**Figure 13:**
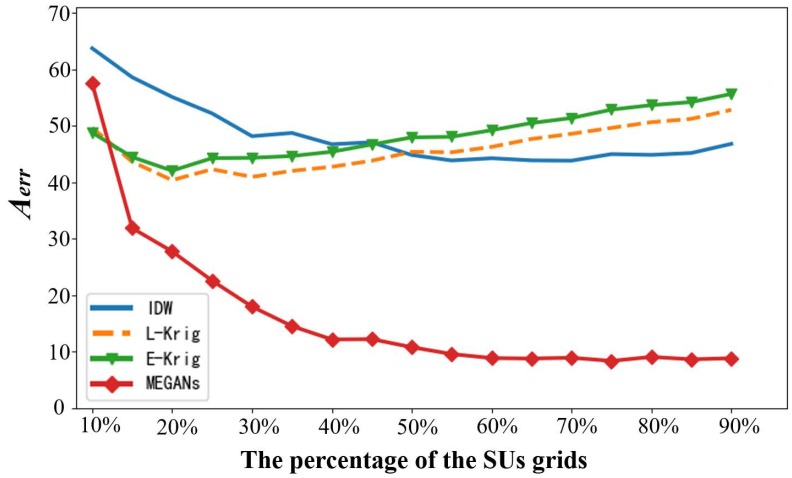
The average image reconstruction errors over different numbers of receiving SUs. The percentages of the SUs’ grids accounting for the whole target region are shown on the horizontal axis. The average image reconstruction errors $A_{err}$ are shown on the vertical axis.

As defined in Section 3.5, the average image reconstruction errors (AIREs) $A_{err}$ measures the gap between the real and reconstructed complete power spectrum maps images. The PSMs are estimated based on the power spectrum measurements from SUs. Therefore, the AIREs are related to the number of receiving SUs. We randomly chose reconstructed PSMs images at 15 different frequency points within the transmitting frequency bands and computed the AIREs of the PSMs images over different numbers of receiving SUs. The simulation result is shown in Figure 13.

As shown in Figure 13, the $A_{err}$ of the MEGANs decreases gradually with the increase of the number of SUs. The MEGANs algorithm outperforms the Kriging method and the IDW interpolation algorithm in the range of 15% to 90% of the SUs’ grids accounting for the whole target region. However, the performance of the proposed algorithm is not good enough at 10% of the SUs’ grids. The above simulation result of MEGANs verifies that the more power spectrum measurements from the receiving SUs, the better the estimation results from the proposed algorithms. The degradation performance of MEGANs at 10% of the SUs’ grids is mainly because there are not enough power spectrum measurements to activate the network of the generator in the forward data propagation process. We can handle this by adjusting the parameters of random-block-pixels measurement function to a smaller value (e.g., α=0.05) in the MEGANs training process. The generator will then get a more powerful reconstruction ability.

As for the $A_{err}$ curve of the IDW interpolation algorithm, it decreases gradually with the increase of the number of SUs, which verifies that the more power spectrum measurements from the receiving SUs, the better estimation results from the IDW algorithm. However, the IDW interpolation algorithm performed the worst of all algorithms tested in the AIREs simulation. The imprecise PSMs estimation result of the IDW algorithm comes from the inaccurate settings of the power value $p_{v}$, which controls the influences of the known nodes on the interpolation node.

Regarding the $A_{err}$ curve of the Kriging interpolation algorithm, the curves of the linear Kriging and exponential Kriging increase after an initial decrease for the reasons:

(a) The measurements data contains little information for the Kriging method in the beginning. The AIREs of the curves decrease from 10% to 20% of the SUs’ grids because of the increase of the measurements data from SUs.

(b) The real distribution of the power spectrum measurements shows a relatively big difference from the imprecise variogram assumptions with the increase of the measurements data from 20% to 90% of the SUs’ grids. The AIREs of the curves rise because the more measurement data from SUs, the larger the deviation between the features of real complex radio environment and the Kriging variogram assumptions.

## 5. Conclusions

In this paper, we proposed a novel power spectrum maps estimation method named maps estimation GANs algorithm for underlay CR networks based on deep learning. On the analogy of auto-encoders and the deep convolutional structure, we designed the generator and the discriminator of the proposed MEGANs to estimate the PSMs and enhance the estimation accuracy. The MEGANs algorithm learns and utilizes accurate radio environment features from the training process, rather than making direct imprecise or biased radio propagation assumptions, as the traditional methods do. Simulation results demonstrate that the MEGANs algorithm provides a more accurate estimation performance than the conventional methods. In our future research, we will focus on the extension of the MEGANs-based power spectrum maps estimation to more practical radio environments; e.g., the shadowing and small-scale fading environment.

## Figures and Tables

**Table 1:** The list of acronyms.

Abbreviation	Full Name
CR	cognitive radio
CRNs	cognitive radio networks
PUs	primary users
SUs	secondary users
QoS	quality of service
PSMs	power spectrum maps
PS	power spectrum
IDW	inverse distance weighted
AI	artificial intelligence
GANs	generative adversarial networks
WGAN-GP	Wasserstein GAN with gradient penalty
MEGANs	maps estimation GANs
RBPa	random-block-patches
RBPx	random-block-pixels
RBPax	random-block-patches-pixels
CNN	convolutional neural network
TV	total variation
AIREs	average image reconstruction errors
Adam	adaptive moment
